# Computational Modelling of Glucose Uptake by SGLT1 and Apical GLUT2 in the Enterocyte

**DOI:** 10.3389/fphys.2021.699152

**Published:** 2021-12-07

**Authors:** Nima Afshar, Soroush Safaei, David P. Nickerson, Peter J. Hunter, Vinod Suresh

**Affiliations:** ^1^Auckland Bioengineering Institute, University of Auckland, Auckland, New Zealand; ^2^Department of Engineering Science, University of Auckland, Auckland, New Zealand

**Keywords:** glucose absorption, nutrient absorption, type 2 diabetes, epithelial transport, CellML, OpenCOR

## Abstract

It has been suggested that glucose absorption in the small intestine depends on both constitutively expressed SGLT1 and translocated GLUT2 in the brush border membrane, especially in the presence of high levels of luminal glucose. Here, we present a computational model of non-isotonic glucose uptake by small intestinal epithelial cells. The model incorporates apical uptake *via* SGLT1 and GLUT2, basolateral efflux into the blood *via* GLUT2, and cellular volume changes in response to non-isotonic conditions. The dependence of glucose absorption on luminal glucose, blood flow rate, and inlet blood glucose concentration is studied. Uptake *via* apical GLUT2 is found to be sensitive to all these factors. Under a range of conditions, the maximum apical GLUT2 flux is about half of the SGLT1 flux and is achieved at high luminal glucose (> 50 mM), high blood flow rates, and low inlet blood concentrations. In contrast, SGLT1 flux is less sensitive to these factors. When luminal glucose concentration is less than 10 mM, apical GLUT2 serves as an efflux pathway for glucose to move from the blood to the lumen. The model results indicate that translocation of GLUT2 from the basolateral to the apical membrane increases glucose uptake into the cell; however, the reduction of efflux capacity results in a decrease in net absorption. Recruitment of GLUT2 from a cytosolic pool elicits a 10–20% increase in absorption for luminal glucose levels in the a 20–100 mM range. Increased SGLT1 activity also leads to a roughly 20% increase in absorption. A concomitant increase in blood supply results in a larger increase in absorption. Increases in apical glucose transporter activity help to minimise cell volume changes by reducing the osmotic gradient between the cell and the lumen.

## 1. Introduction

Glucose absorption *via* the small intestine is the primary source of metabolisable energy in humans and other animals. The process of glucose absorption has been studied for many years (McCance and Madders, 1930; Wertheimer, 1934); however, there remain open questions about the pathways and transporters involved in different species and under different conditions (Karasov, 2017).

Until about two decades ago, SGLT1 had been known as the only glucose absorption pathway in the cell (Ferraris, 2001). The classical model of intestinal glucose absorption is adequate when the glucose concentration in the lumen is low. However, SGLT1 is saturated around 30 mM of glucose whereas glucose absorption is not saturated even at 100 mM of extracellular glucose (Kellett and Helliwell, 2000) and instead is similar to simple diffusion (Fullerton and Parsons, 1956; Debnam and Levin, 1975). The responsible mechanism for the diffusive process of glucose absorption has been a matter of debate. One of the first theories was a paracellular flow or solvent drag which was based on the association of glucose absorption with water absorption (Fullerton and Parsons, 1956; Pappenheimer and Reiss, 1987). According to this theory, SGLT1-mediated absorption of glucose from lumen to the cell and efflux into blood builds up an osmotic gradient for water absorption which allows transport of glucose and other nutrients through the tight junction along with water (Pappenheimer, 1987; Pappenheimer and Reiss, 1987). This theory was controversial since a number of studies found that paracellular glucose absorption is negligible (Ferraris et al., 1990; Ferraris and Diamond, 1997; Lane et al., 1999). Following the detection of GLUT2 in the brush border membrane of the diabetic rat (Corpe et al., 1996) another theory has been proposed according to which the recruitment of GLUT2 to the apical membrane of epithelial cell in case of high luminal glucose provides the additional absorptive capacity observed in experiments (Kellett and Helliwell, 2000; Au et al., 2002; Affleck et al., 2003; Gouyon et al., 2003). Imaging studies have also shown translocation of GLUT2 from the basolateral to the apical membrane as a rapid, reversible response to a glucose stimulus (Cohen et al., 2014). Other studies have suggested that short term upregulation of SGLT1 may play a role in increasing glucose absorption in response to a stimulus (Sharp et al., 1996; Cheeseman, 1997; Stearns et al., 2010; Koepsell, 2020). Thus, the mechanisms by which enterocytes can rapidly increase absorption in response to a glucose stimulus are still a matter of controversy. In this situation, mathematical models will be useful for interpreting data and exploring the implications of the different hypotheses.

A model of sodium/potassium homeostasis in the enterocyte during SGLT1-mediated absorption was developed by Thorsen et al. (2014). This model was limited to iso-osmotic conditions and did not consider short term changes in apical glucose transporters in response to a glucose stimulus. Maintenance of cell volume is very important for the appropriate function of the body. Cell volume regulation usually depends on the transport of the solute to the extracellular compartment and cell permeability to water. The main driver of cell volume change is the change in osmolarity. The osmolarity difference between the cell and the extracellular compartment can cause the cell to swell by gaining water or shrink by losing water (Argyropoulos et al., 2016). Glucose transport across the cell can change the osmolarity leading to changes in water transport and cell volume. For instance, glucose loaded in the lumen can increase the osmolarity of the lumen over that of the cell thus causing more water to leave the cell and making the cell volume smaller (Zeuthen et al., 2007).

Another model examined the role of apical GLUT2 in sugar absorption, water transport, and cell volume regulation (Naftalin, 2014). Glucose absorption by SGLT1 causes the intracellular glucose concentration to exceed the luminal concentration. Consequently, the cytosol becomes hyperosmolar leading to a large increase in cell volume. The presence of GLUT2 in the apical membrane causes glucose to leak back into the lumen out of the cell, reducing intracellular osmolarity and moderating volume changes. In this model, the primary pathway for glucose absorption is apical SGLT1 and basolateral GLUT2, with apical GLUT2 mainly serving as an osmoregulator.

In the current model, we extended our previous work (Afshar et al., 2019) to include non-isotonic transport and developed a validated model of glucose uptake in the small intestine based on a mechanistic model for all relevant transporters. While similar in approach to the Naftalin (2014) model, the inclusion of individual transporters allowed us to keep track of all ionic species (Na^+^, K^+^, Cl^−^, and H^+^) and investigate the effect of changing the activity of individual transporters. This integrative model is implemented in CellML and simulated using OpenCOR environment. We adopted a modular compositional approach to construct the model.

## 2. Methods

### 2.1. Model Construction

**Figure 1:**
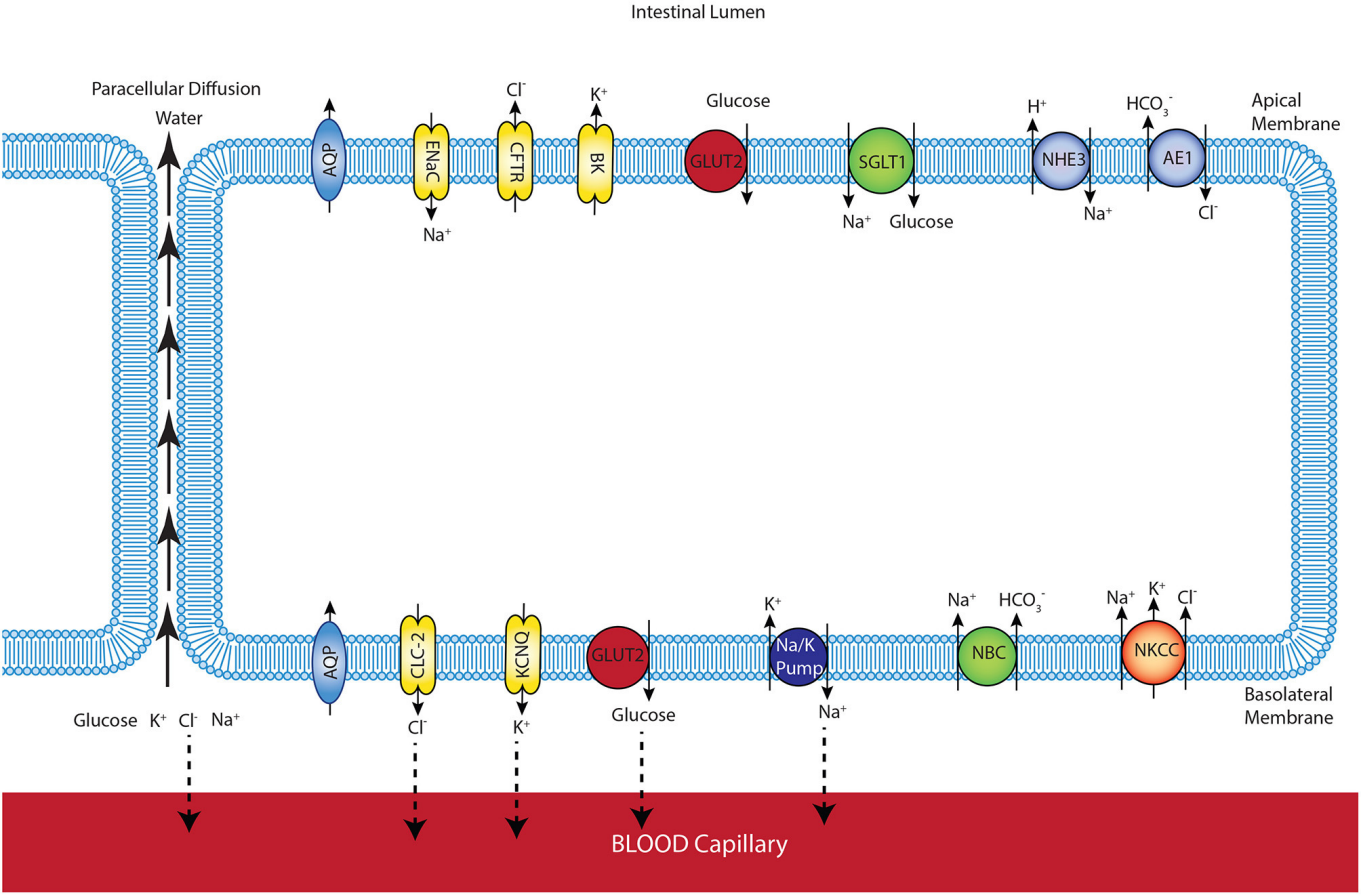
Schematic representation of enterocyte showing the transporters relevant to glucose transport in the apical and basolateral membranes along with the apical (lumen) and basolateral (interstitium) extracellular domains.

In our previous study, the mathematical model of glucose uptake by the enterocyte was constructed and included the relevant transporters from the literature (Barrett and Keely, 2015). The model was isotonic through all compartments and water movement through the apical and basolateral membranes or paracellular pathways was not considered. Here, we extended the model by adding water transport and considered the cell volume changes during the absorption process. Figure 1 shows a schematic diagram of a 3-compartment model of an epithelial cell in the small intestine with the relevant transporters for glucose absorption.

The model allows us to predict membrane potentials, intracellular concentrations of glucose and electrolytes (Na^+^, K^+^, ${\text{HCO}}_{3}^{-}$, Cl^−^), and the fluxes of these species. The composition and volume of the cell compartment are not fixed and can change due to water absorption from or secretion into the extracellular compartments. The mucosal (intestinal lumen) compartment was treated as an infinite bath of constant composition. The blood (serosal) compartment has a finite, fixed volume. To maintain a fixed serosal volume, outlet blood flow was variable and equal to a constant inlet blood flow plus water flux across the basolateral cell membrane. Ion and glucose concentrations in this compartment are also variable and determined by transcellular fluxes across the basolateral membrane, paracellular fluxes between the cells, and the blood flow rate. Transcellular and paracellular water movement is based on the osmolarity difference between the two involved compartments, which leads to changes in cell volume. Flux balance and electric charge conservation laws yield the governing equations of the model.

All equations for ions' fluxes and concentrations are described in the Supplementary Material. The models and their associated parameters can be found at the following link: https://models.cellml.org/workspace/58c.

### 2.2. SGLT1 and GLUT2 Models

**Figure 2:**
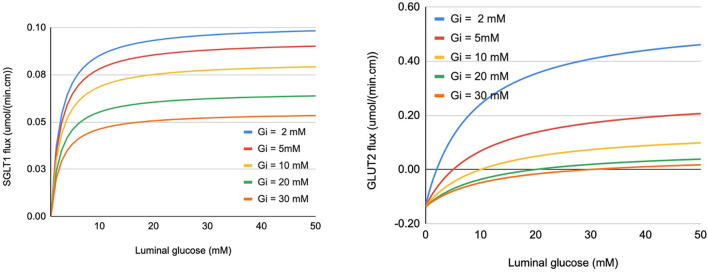
Glucose flux through SGLT1 (left panel) and apical GLUT2 (right panel) under varying conditions of luminal and intracellular glucose (Gi). Fluxes are expressed per cm of intestinal length using parameters specified in section 2.3.

Glucose flux through SGLT1 was described using a 6-state kinetic model for SGLT1 (Parent et al., 1992) with parameter values from Wright et al. (2011). An alternating conformation model was used for GLUT2 with parameter values from Lowe and Walmsley (1986). Glucose flux through the transporters for a range of extra- and intracellular glucose concentrations is shown in Figure 2. The SGLT1 flux was calculated with constant values of the apical membrane potential (−65 mV) and luminal sodium (150 mM). Varying these values had minimal impact on the flux. Equivalent *V*_*max*_ and *K*_*m*_ values were determined by fitting a steady Michaelis-Menten expression to each line in the plots (Table 1). The values are consistent with those reported in the reviews by Karasov (2017) and Koepsell (2020). The *V*_*max*_ and *K*_*m*_ values are derived quantities not directly used in the modelling here, but provided a check that the model parameter values are reasonable.

**Table 1:** *V*_*max*_ and *K*_*m*_ values for SGLT1 and GLUT2 determined by fitting to the flux values in Figure 2.

	**SGLT1**		**GLUT2**	
**Intracellular glucose (mM)**	**V_***max***_ [μmol/(min.cm)]**	**K_***m***_ (mM)**	**V_***max***_ [μmol/(min.cm)]**	**K_***m***_ (mM)**
2	0.104	1.8	0.577	12
5	0.098	1.85	0.274	15
10	0.081	1.85	0.144	20
20	0.064	1.85	0.072	30
30	0.053	1.85	0.058	43

### 2.3. Model Validation

The model was validated against the published intestinal loop data of the uptake of sodium, glucose, and water measured in dogs (Collin et al., 1978). In the experiments, a 75 cm loop of intestine was isolated and infused luminally at a rate of 2.9 ml/min with a solution containing 100 mM glucose and 90 mM sodium chloride. Measurements of water, sodium, and glucose fluxes were reported. The composition of the infusate was used as the fixed mucosal concentration in the model. Concentrations of ions in the inflowing blood were kept close to the literature values (Table 2).

**Table 2:** Reported values for ions, concentrations in blood.

**Ion**	**Blood concentration (mM)**	**Reference**
Gl	< 7.8	NIfHaC, 2012
Na^+^	135–145	Pierro et al., 2012
K^+^	3.5–5.5	Posencheg and Evans, 2012
Cl^−^	97–107	Pfortmueller et al., 2018

For the model parameters, we estimated a range based on information in the literature. Based on the anatomy of the dog's small intestine (diameter 1 cm, number of villi 23 per mm^2^) (Kararli, 1995), villus dimensions (height 0.75 mm, diameter 0.4 mm) (Kuzmuk et al., 2005), and the typical surface area of an enterocyte (42 μm^2^) (Thorsen et al., 2014), the number of epithelial cells in the experimental segment was estimated to be 1.3 × 10^9^. Using an estimate of 35–122 ml/(100 g · min) for the intestinal blood flow rate (Granger et al., 1980), the flow to the blood compartment of each cell is 1–4 ×10^−17^ m^3^/s. The blood volume of the mucosa is around 4.7 ml/100 g (Rieke and Everett, 1957), and the mass of mucosa is about 0.35 g/cm of the intestine (Robinson et al., 1977). By dividing the volume by the number of cells, the volume of the blood compartment for each epithelial cell is 1–4 ×10^−16^m^3^.

Baseline values for the blood volume and perfusion rate per cell were set at 1 × 10^−16^ m^3^ and 1 × 10^−17^ m^3^/s in this work. In addition, the blood volume is held constant by setting the outlet blood flow to be equal to the sum of the inlet blood flow and water flux across the basolateral membrane.

## 3. Results

### 3.1. Model Response to Glucose Stimulus

We first examined the response of the model to a step change in luminal glucose concentration. For these simulations, ions' concentrations in the luminal and blood compartments were initialised at 150 mM Na^+^, 5 mM K^+^, and 150 mM Cl^−^. The luminal compartment was considered an infinite bath (constant ion concentration) while the volume of the blood compartment was treated as finite and ions' concentrations were allowed to vary. Luminal glucose was fixed at zero for *t* = 0–5,000 s and changed to 50 mM for *t*= 5,000–10,000 s. Inflowing blood glucose was fixed at 4 mM for the entire simulation. The aim of these simulations is to test the stability of the model equations and the numerical scheme, i.e., to check if they reached reasonable steady state values in response to a perturbation in the initial conditions. They should not be taken as indicative of a physiological response.

**Figure 3:**
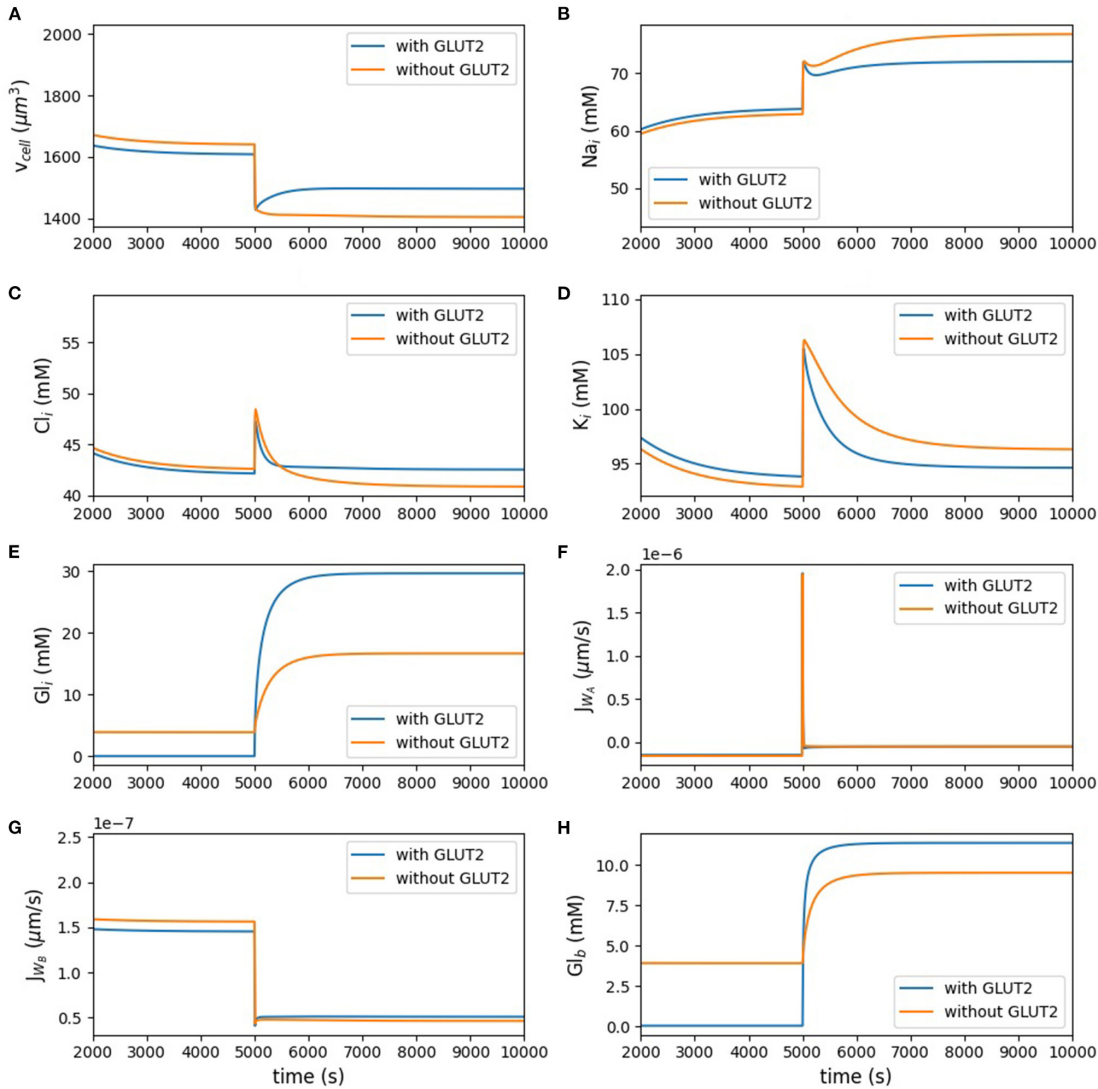
Model behaviour in the absence of luminal glucose (*t* < 5, 000 s) and following a step change to 50 mM luminal glucose (5, 000 ≤ *t* < 10, 000 s) of the simulation. **(A)** cell volume; **(B)** intracellular concentrations of sodium; **(C)** chloride; **(D)** potassium; **(E)** glucose; **(F,G)** apical and basolateral water flux; **(H)** blood glucose.

Figure 3 and Table 3 show the dynamic response and steady state values of ions and glucose concentrations, cell volume, and water fluxes in the absence and presence of GLUT2 in the apical membrane.

**Table 3:** Steady state model predictions with and without apical GLUT2 with luminal glucose = 50 mM compared to literature values.

**Variable**	**Model with no apical GLUT2**	**Model with apical GLUT2**	**Reported value**	**Reference**
Na^+^ (mM)	63	73	45−65	Nellans and Schultz, 1976
K^+^ (mM)	93	96	120−140	Nellans and Schultz, 1976
Cl^−^ (mM)	43	42	50−70	Frizzell et al., 1973; Nellans et al., 1973
Apical (lumen-cell) membrane potential (mV)	−38	−36	−36 ± 0.5	Rose and Schultz, 1971
Basolateral (interstitium-cell) membrane potential (mV)	−51	−51	−40.5 ± 0.8	Rose and Schultz, 1971
pH	7.1	7.05	7.2	Shimada and Hoshi, 1987

*Number of transporters per cell: SGLT1 = 3 × 10^7^, apical GLUT2 = 10^8^, basolateral GLUT2 = 2 × 10^8^*.

Figure 3A represents the volume of the cell. Figures 3B–D show the intracellular concentration of sodium, chloride, potassium, and glucose. The other panels in Figure 3 present the water flux through the apical and basolateral membranes, and the last panel depicts the glucose concentration in the blood. Change of the cell volume after applying the stimulus is less in the presence of apical GLUT2. Potassium and chloride concentrations did not change much by having glucose stimulus in the lumen, but sodium concentration increased after applying the glucose stimulus; however, all the aforementioned concentrations did not considerably change in the presence of apical GLUT2. Intracellular glucose concentration with the GLUT2 transporter located in the apical membrane increased more under the luminal stimulus, which shows that more glucose entered the cell through apical GLUT2. Water fluxes across the apical and basolateral membranes both showed a very rapid response to the stimulus and they both reached the same value at the steady state.

The blood glucose concentration (Figure 3H) before the luminal stimulus is lower in the presence of apical GLUT2. In this case, since there is a constant influx of glucose into the blood compartment (inflowing blood at 4 mM), basolateral and apical GLUT2 provide a pathway for glucose to move from the blood to the lumen. Since the blood compartment has a finite volume, this leads to a rapid depletion of glucose both from the cell (Figure 3E) and blood. Without apical GLUT2, there is no pathway for glucose to enter the lumen (reverse transport through SGLT1 is not considered) and hence concentrations in the cell and blood compartment equalise around 3.9 mM. When the luminal glucose concentration is raised to 50 mM, uptake by apical GLUT2 and/or SGLT1 and efflux by basolateral GLUT2 causes a rise in the blood glucose levels. This is more pronounced in the presence of apical GLUT2.

### 3.2. Comparison With Intestinal Loop Data

**Figure 4:**
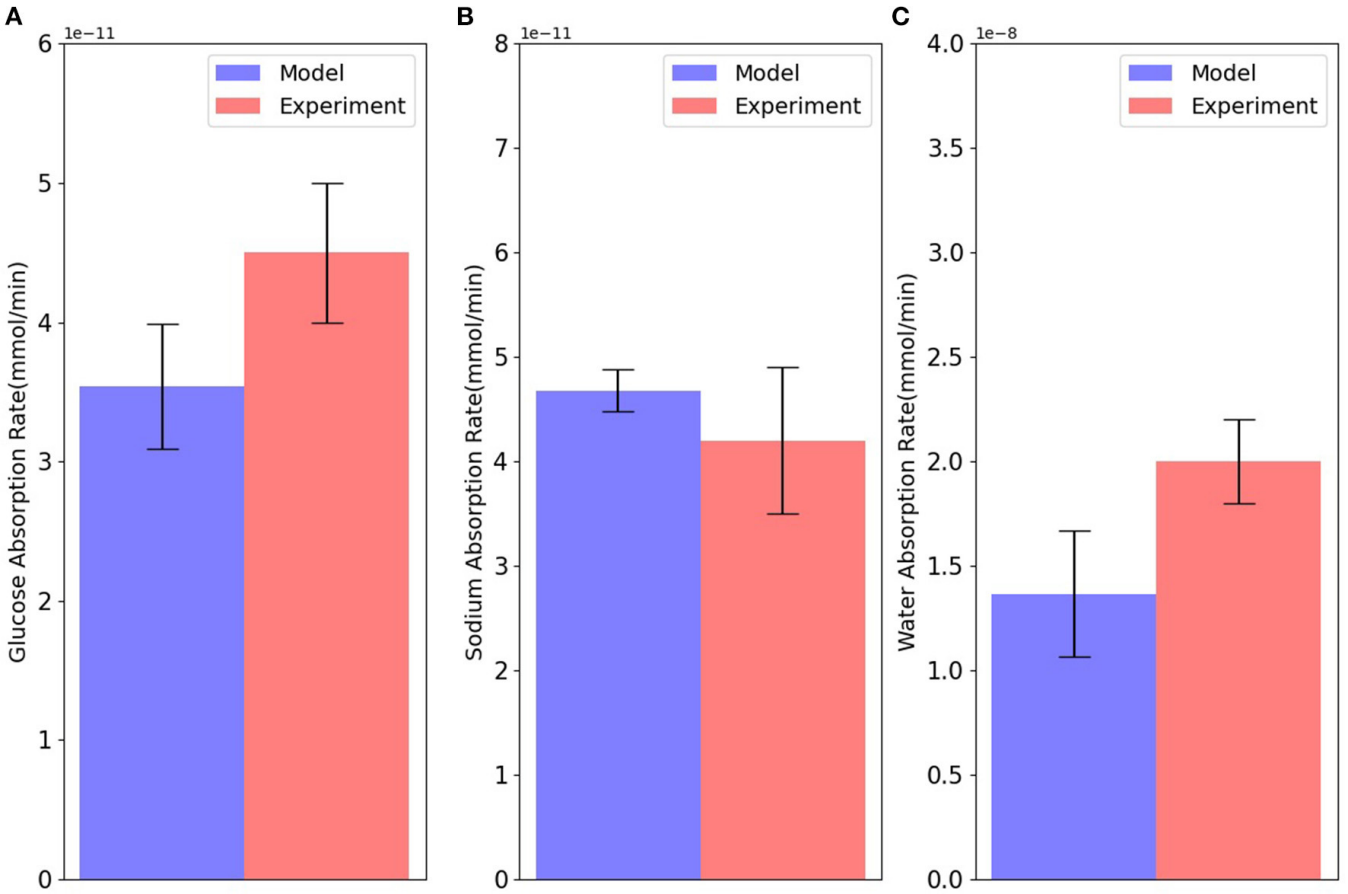
Absorption rates of **(A)** glucose; **(B)** sodium; and **(C)** water through the cell in comparison with intestinal loop data. Error bars in the model represent different values for inlet blood flow. Experimental bars are mean ± SE with 6 tests (Collin et al., 1978).

Next, model predictions were compared with measurements from an intestinal loop study carried out in dogs (Collin et al., 1978). Since the experiments used a continuous infusion of the mucosal surface, the lumen concentrations in the simulations were fixed at the experimental values (90 mM NaCl, 100 mM glucose, and 6 mM potassium). The number of apical GLUT2 and basolateral GLUT2 were both fixed at 2 × 10^9^, and the number of SGLT1 at 1 × 10^8^. As there was not enough information about blood volume or inlet blood flow in the experiment, we considered three different values for inlet blood flow (6 × 10^−18^, 1 × 10^−17^, and 3 × 10^−17^ m^3^/s) to account for uncertainty in the actual value of inlet blood flow. Figure 4 shows that the steady state model predictions are in reasonable agreement with the intestinal loop data. The mismatch in the absorption rates of glucose, water, and sodium is around 20–25%, which is within the uncertainty of the measurements and model, indicated by the error bars in the figure.

### 3.3. Model Response to Varying Luminal Glucose

**Figure 5:**
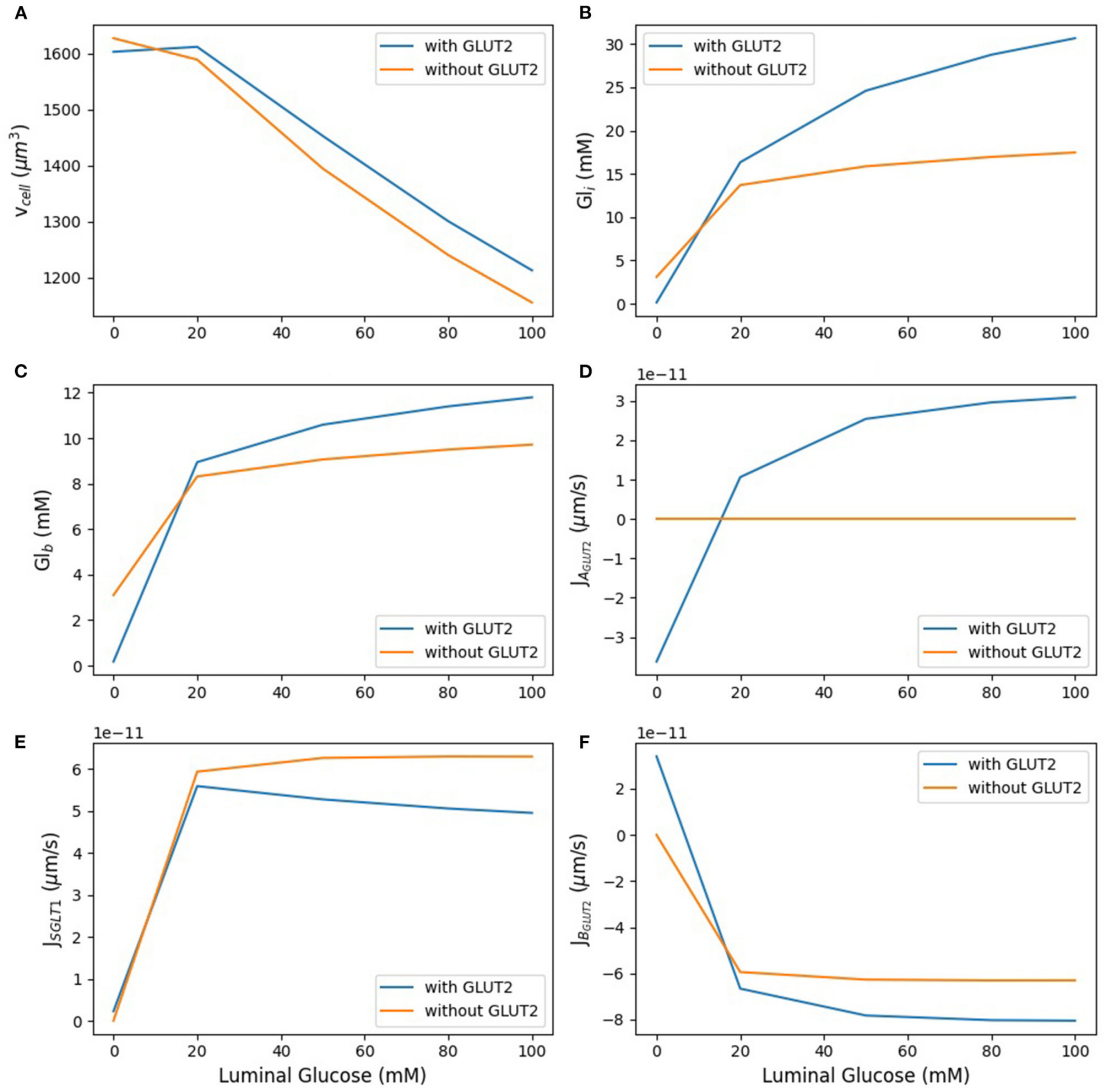
Model response to different luminal glucose concentration. **(A)** Cell volume; **(B)** intracellular glucose; **(C)** blood glucose; **(D)** apical GLUT2 flux; **(E)** SGLT1 flux; **(F)** basolateral GLUT2 flux. The simulation was run under five different luminal glucose concentration (0, 20, 50, 80, and 100 mM). Simulation parameters: number of apical GLUT2 = 10^8^, number of basolateral GLUT2 = 2 ×10^8^, number of SGLT1 = 3 ×10^7^, inflow blood glucose = 4 mM, inlet blood flow rate = 10^−17^ m^3^/s, and blood volume = 10^−16^ m^3^.

In the next step, we examined the effect of apical GLUT2 on the steady state response of the model as the luminal glucose concentration was increased from 0 to 100 mM. In Figure 5, the contribution of apical GLUT2 to different glucose concentrations and glucose fluxes is shown. There is a clear distinction between the behaviour at zero luminal glucose and at luminal glucose values ≥ 20 mM. We first discuss the response for luminal glucose in the range 20–100 mM.

As luminal glucose increases from 20 to 100 mM, the osmolarity of the luminal bath increases and leads to water efflux from the cell and hence a decrease in cell volume (Figure 5A). This decrease is lower in the presence of apical GLUT2 since glucose absorption through this transporter leads to higher intracellular glucose concentrations (Figure 5B) and lower osmolarity differences in the lumen. The increase in intracellular glucose is significantly different between the two scenarios: 82% with apical GLUT2 whereas without apical GLUT2 it increases only 25%. There is a concomitant increase in the blood glucose concentration (Figure 5C), although the magnitude is smaller (25% in the presence of apical GLUT2 vs. 12% in the absence). Figures 5D,E show apical glucose flux through GLUT2 and SGLT1. As expected, the GLUT2 flux increases with luminal glucose. The increase is by a factor of 3 over the concentration range and levels off at higher concentrations, showing that the transporter is close to saturation (Figure 5D). The SGLT1 flux is 1.5-fold to 5-fold larger than the GLUT2 flux over the entire concentration range (Figure 5E). The difference as well as the absolute SGLT1 flux decreases with increasing luminal glucose. The decrease in absolute flux is small (roughly 10%) and can be explained by the lower glucose concentration difference between the lumen and the cell in the presence of apical glucose. This decrease leads to a small reduction in the driving force for SGLT1 mediated transport. Basolateral GLUT2 flux is higher in the presence of apical GLUT2 due to the higher intracellular—blood glucose concentration gradient (Figure 5F), which increases from about 8–18 mM as luminal glucose concentration is increased. Note that the negative value of the basolateral flux indicates efflux from the cell. The flux difference in the two scenarios increases from about 10 to 30% with increasing luminal glucose.

In the absence of luminal glucose, there is a blood-to-lumen glucose concentration gradient and the presence of apical GLUT2 provides a pathway for glucose transport from the blood to the lumen. The signs of the apical and basolateral GLUT2 flux are reversed from before, indicating influx into the cell from blood and secretion into the lumen. Blood and intracellular glucose concentrations equilibrate to values close to zero. Taken together, the results indicate that the presence of apical GLUT2 increases glucose absorption and blood glucose concentrations when luminal glucose concentrations are high, but leads to glucose efflux from the blood to lumen at luminal glucose is low.. Enhancement in absorption is small (roughly 10%) at luminal glucose concentrations of 20 mM and increase to about 30% at luminal glucose of 100 mM. The effect of apical GLUT2 on cell volume is small, the presence of apical GLUT2 reduces water loss from the cell by less than 10% over the concentration range.

In these simulations, model parameters such as transporter density, blood flow rate, and inlet blood glucose were held constant. The following sections explore the effect of varying these parameters.

### 3.4. Effect of Varying Transporter Density

**Figure 6:**
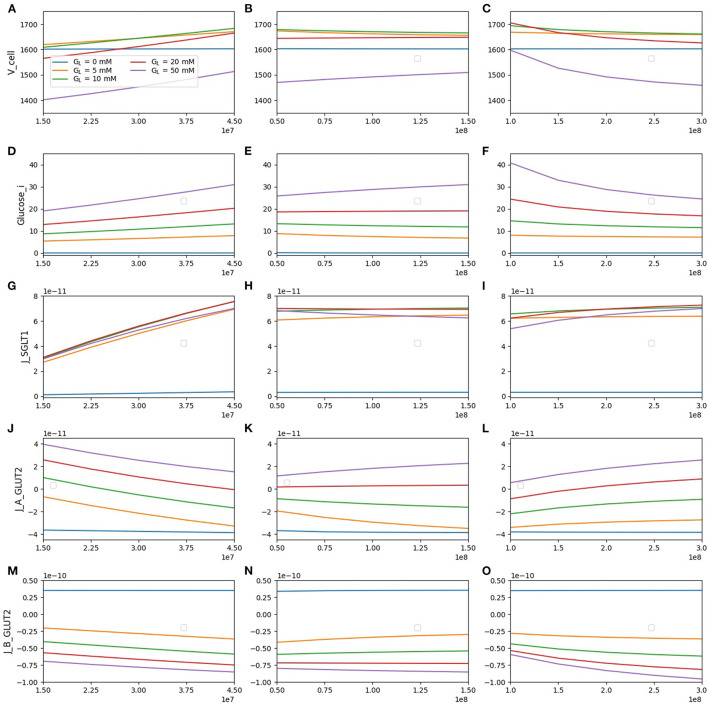
Model response to different transporter densities. **(A–C)** Cell volume; **(D–F)** intracellular glucose; **(G–I)** SGLT1 glucose flux; **(J–L)** apical GLUT2 flux; **(M–O)** basolateral GLUT2 flux. In each column, one transporter density is varied while the other two are held constant at the reference value [nSGLT1 = 3 × 10^7^, nGLUT2 (apical) = 10^8^, nGLUT2 (basolateral) = 2 × 10^8^].

The densities of SGLT1, apical GLUT2, and basolateral GLUT2 were all varied between 0.5 and 1.5 times the baseline value and the effects on steady state values of cell volume, intracellular glucose, and glucose fluxes were examined. In each case, the density of one transporter was varied, while the other two were fixed at the reference value. Results are shown in Figure 6 for luminal glucose concentrations varying from 5 to 50 mM.

The cell volume (top row) increases with SGLT1 density for all luminal glucose concentrations. In contrast, an increase in apical GLUT2 density causes cell volume to increase only for a luminal glucose concentration of 50 mM. An increase in basolateral GLUT2 leads to a decrease in cell volume. These results can be explained by the changes in intracellular glucose concentration (second row). An increase in SGLT1 density causes an increase in intracellular glucose at all luminal glucose values, whereas an increase in apical GLUT2 causes an increase only in luminal glucose = 50 mM. Changes in cell osmolarity and hence cell volume mirror this change in intracellular glucose concentration.

Glucose flux through SGLT1 (third row) increases by about 2.5-fold as the SGLT1 density increases 3-fold. This increase is quite insensitive to the luminal glucose concentration. The SGLT1 flux did not change significantly (< 25%) with the apical or basolaterial GLUT2 density. The change is largest at high luminal glucose: an increase in apical GLUT2 increases intracellular glucose reducing the effective *V*_*max*_ of SGLT1 (Table 1), whereas an increase in basolateral GLUT2 has the opposite effect.

Flux through apical GLUT2 (fourth row) shows some interesting features. At the two lowest luminal glucose concentrations (5 and 10 mM), the flux is negative (i.e., glucose exits the cell through apical GLUT2) since intracellular glucose is higher than luminal glucose. It decreases with increasing SGLT1 density, which is a result of the reduction in the luminal-intracellular concentration gradient due to SGLT1-mediated glucose entry. It increases with an increase in basolateral GLUT2 density as more glucose is removed across the basolateral membrane.

At the basolateral membrane, glucose can enter or leave the cell *via* GLUT2 depending on the concentration gradient. For luminal glucose concentrations ≥ 5mM and inlet blood glucose of 4 mM, flux across the basolateral GLUT2 (bottom row) is always negative (glucose leaves the cell). Net glucose absorption (magnitude of the flux) increases with SGLT1 density and luminal glucose concentration. It increases with apical GLUT2 only at high luminal glucose concentrations. At 5 and 10 mM luminal glucose, an increase in apical GLUT2 leads to a decrease in glucose uptake since the cell-blood concentration gradient is reduced. Moreover, in the absence of luminal glucose, the concentration gradient changes sign, and glucose enters the cell from the blood (positive flux) and then is secreted into the lumen *via*Increasing apical GLUT2 leads apical GLUT2.

The results suggested that an increase in apical SGLT1 is most effective at increasing glucose uptake, even at high luminal glucose concentrations (> 15% increase at 50 mM luminal glucose as SGLT1 density trebled). Increasing apical GLUT2 leads to increasing uptake by about 10% for the same conditions. At low luminal glucose (5 and 10 mM), an increase in apical GLUT2 leads to a decrease in glucose uptake due to glucose secretion from the cell to the lumen.

### 3.5. Effect of Blood Flow Rate and Inlet Blood Glucose

**Figure 7:**
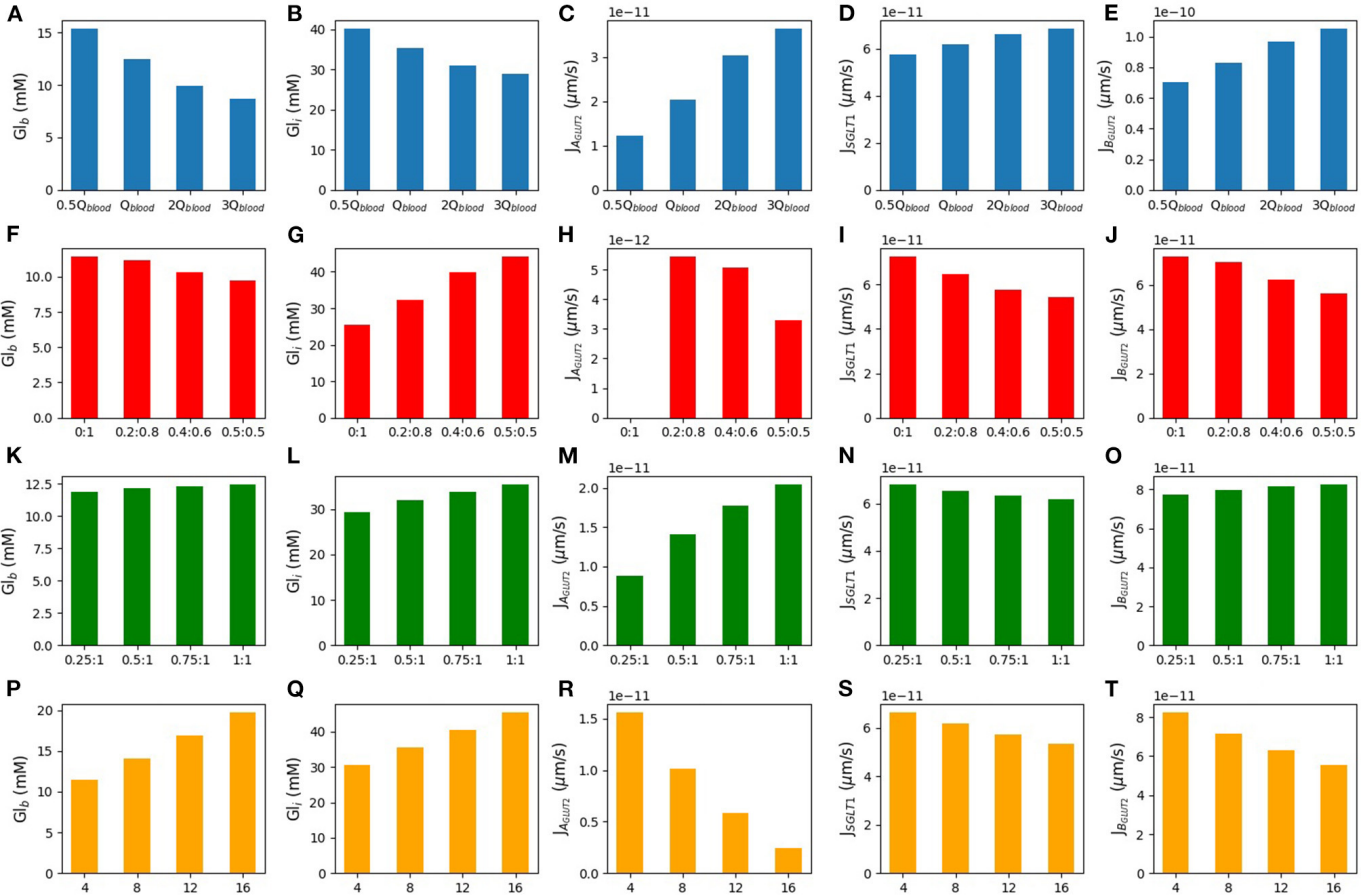
Blood glucose concentration, intracellular glucose concentration, apical GLUT2 flux, SGLT1 flux, and basolateral GLUT2 flux under different conditions. The first row is the model response to different blood flow rates. ${\text{Q}}_{blood}=10^{-17}$ m^3^/s is the baseline value of the blood flow rate **(A–E)**. The second row shows the response to variations in the inlet blood glucose concentration **(F–J)**. Rows three and four consider two different scenarios for GLUT2 translocation to the apical membrane. Row three shows simulations run under the assumption that apical GLUT2 is translocated from basolateral GLUT2. Different ratios between apical and basolateral GLUT2 are considered with the total number fixed **(K–O)**. Row four shows simulations run under the assumption that apical GLUT2 is translocated from intracellular vesicles. The number of basolateral GLUT2 is fixed and labels represent the fraction of total GLUT2 in the apical and basolateral membranes **(P–T)**.

In Figure 7, we consider the effect of varying inlet blood flow and blood glucose concentration for the model with apical GLUT2 and 50 mM glucose in the lumen. The first row of Figure 7A shows that increasing the blood flow from half of the baseline blood flow to 3 times higher, leads to a decrease in both blood glucose (16–8 mM) and intracellular glucose concentrations (40–28 mM). The decrease in intracellular glucose increases the driving force for absorption *via* apical GLUT2, leading to a 3-fold increase in the apical GLUT2 flux (Figure 7C). There is also a small increase in the SGLT1 flux of about 15% (Figure 7D). The total glucose flux into the cell is dominated by SGLT1 (Figure 7E, 5-fold to 1.5-fold larger than apical GLUT2 flux) over the entire range of flow rates. The increase in apical flux *via* GLUT2 and SGLT1 translates to a 60% increase in net basolateral flux (i.e., glucose uptake) as blood flow increases. Roughly, two-thirds of this increase can be attributed to apical GLUT2 and one-third to an increase in SGLT1 flux.

Next, the effect of increasing the inlet blood glucose concentration in the range of 4–16 mM was considered (second row). As expected, the blood compartments (outlet) glucose concentration and cell concentration both increase by about 70 and 40%, respectively. The increase is almost linear. There is a concomitant decrease in flux through the apical and basolateral transporters. Apical GLUT2 flux reduces 8-fold, while SGLT1 flux reduces by about 20% and basolateral flux by about 30%. Roughly, 60% of the decrease can be attributed to the reduction in apical GLUT2 flux and the rest to SGLT1.

The results suggest that the contribution of apical GLUT2 to glucose uptake is quite sensitive to inlet blood flow and glucose concentrations. Apical GLUT2 is most effective at increasing uptake when the inlet blood concentrations are low and the flow rate is high. However, the steady state apical GLUT2 flux varies about 15–20 fold between the extremes of the conditions considered here. On the other hand, SGLT1 flux is less sensitive to variations (about 20% difference between the extremes) and is also larger than the apical GLUT2 flux by 2–20-fold over the range of inlet blood flow and concentrations studied.

### 3.6. Different Modes of GLUT2 Translocation

Here, we considered two different sources of apical GLUT2. In the first scenario, the source of apical GLUT2 is considered to be translocation from intracellular vesicles or newly synthesised protein. Therefore, the number of basolateral GLUT2 is fixed at 2 × 10^8^, and the number of apical GLUT2 varies from 0.25- to 1-fold of basolateral GLUT2. Results are shown in the third row of Figure 7. Increasing the number of apical GLUT2 leads to higher apical GLUT2 flux (Figure 7M) and higher intracellular concentration (Figure 7L). Basolateral flux (Figure 7O) and blood concentration (Figure 7K) increase by a small quantity (14 and 10%, respectively). SGLT1 flux shows about 10% decrease as apical GLUT2 increases (Figure 7N).

In the second scenario, we considered the translocation of basolateral GLUT2 to the apical membrane in response to a glucose stimulus. Thus, the total number of GLUT2 is held fixed at 2 × 10^8^ and is all located on the basolateral membrane when the luminal glucose is zero. When a glucose stimulus of 50 mM is applied, a fraction of these (0.2, 0.4, or 0.5) translocate to the apical membrane. The fourth row of Figure 7 shows the steady state model response to these conditions. The X-axis shows the apical and basolateral density of GLUT2 as a fraction of the total GLUT2 density. In this case, increasing the number of GLUT2 in the apical membrane leads to less basolateral GLUT2 and then less glucose is expected in the blood (20% decrease in blood glucose concentration-Figure 7P). Increasing apical GLUT2 increases the intracellular glucose concentration from 25 to 45 mM (80%-Figure 7Q). Figure 7R shows the apical GLUT2 flux, which decreases as the fraction of translocated GLUT2 increases from 0.2. This is a result of the decrease in the lumen-cell glucose gradient as the intracellular concentration increases. The same effect leads to a decrease in the SGLT1 flux by about 20% (Figure 7S). Since the SGLT1 flux is about 10 times higher than the apical GLUT2 flux, this translates to a reduction in glucose flux to blood (Figure 7T).

Together, the results showed that an increase in apical GLUT2 expression alone does not imply greater glucose absorption. If the source of apical GLUT2 is translocation from the basolateral membrane, it can lead to a decrease in net absorptive flux and blood glucose levels. Even if apical GLUT2 is sourced from intracellular vesicles or newly synthesised, the increase in absorption is small. These results are related to an increase in intracellular glucose and a decrease in SGLT1 flux.

### 3.7. Short Term Regulation of SGLT1

The results thus far focused on dynamic upregulation of apical GLUT2 without modulating SGLT1 expression and indicated the kinetic responses of SGLT1 flux serve to counteract changes in glucose uptake resulting from changes in luminal glucose, blood flow rates, and inlet blood concentration. Next, we examined how simultaneous short-term increases in SGLT1 and apical GLUT2 expression in response to a glucose stimulus affect absorption. Five scenarios were considered: baseline with no apical GLUT2 (A), 50% increase in SGLT1 and apical GLUT2 equal to 0.5 times basolateral GLUT2 (B), baseline SGLT1 and apical GLUT2 equal to 1.0 times basolateral GLUT2 (C), 100% increase in SGLT1 and apical GLUT2 equal to 0.5 times basolateral GLUT2 (D), and 100% increase in SGLT1 alone with no apical GLUT2 (E). Luminal glucose concentration was fixed at 50 mM.

**Figure 8:**
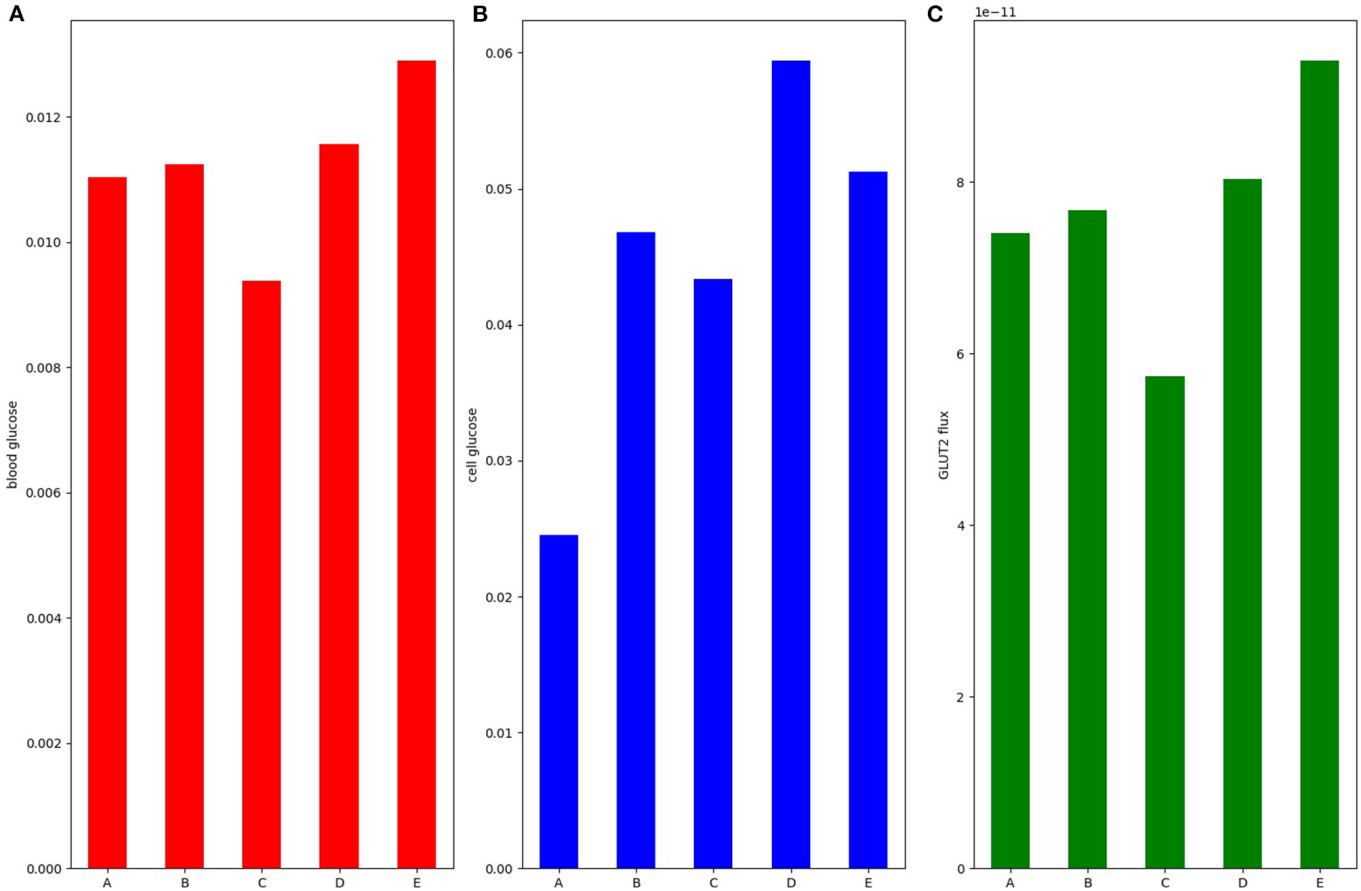
**(A)** Blood glucose; **(B)** intracellular glucose; and **(C)** glucose flux into the blood. Simulation was run under 50 mM stimulus of luminal glucose. Bar A: baseline with no apical GLUT2. Bar B: 50% increase in SGLT1 and apical GLUT2 equal to 0.5 times basolateral GLUT2. Bar C: baseline SGLT1 and apical GLUT2 equal to 1.0 times basolateral GLUT2. Bar D: 100% increase in SGLT1 and apical GLUT2 equal to 0.5 times basolateral GLUT2. Bar E: 100% increase in SGLT1 alone with no apical GLUT2.

At baseline, cell and blood glucose concentrations are about 25 and 11 mM, respectively (Figure 8A). A 50% increase in SGLT1 (Figure 8B) or a 1:1 ratio of apical to basolateral GLUT2 (Figure 8C) both lead to more glucose inside the cell (48 and 43 mM, respectively). Blood glucose flux and concentration increase slightly in B but reduce in C since the efflux capacity at the basolateral membrane is decreased. In conditions D (100% increase in SGLT1, 0.5:1 apical to basolateral GLUT2) and E (100% increase in SGLT1, no apical GLUT2), the intracellular concentration goes higher than lumen. The intracellular concentration in D is higher and the blood concentration and flux lower than E due to the reduced efflux capacity at the basolateral membrane. The results indicate that an increase in SGLT1 expression is the most effective means of increasing glucose absorption.

## 4. Discussion

We developed a mathematical model to examine how short-term increases in SGLT1 and apical GLUT2 in response to a glucose stimulus influences glucose absorption and volume regulation in an enterocyte exposed to a hyperosmotic lumen. The results indicated that in the absence of apical GLUT2, the cell shrinks by about 25% as luminal glucose increases from 0 to 100 mM and that apical GLUT2 reduces this shrinkage to about 20%. The effect on glucose absorption is more complex and depends on the luminal glucose concentration, blood flow rates, and blood glucose concentrations, as well as the source of the apical GLUT2. At low luminal glucose, the presence of apical GLUT2 reduces net absorption. For luminal glucose concentrations of 0, 5, and 10 mM, the presence of apical GLUT2 leads to glucose being transported out of the cell into the lumen. When luminal glucose concentrations are lower than blood glucose, basolateral and apical GLUT2 provide a pathway for the passive secretion of glucose from the blood to the lumen. Similar results would apply for any passive apical glucose transporter, for example GLUT5. While it is generally considered that GLUT5 does not transport glucose (e.g., studies reviewed in Koepsell, 2020), *in vitro* studies have shown low levels of D-glucose transport by GLUT5 (Nomura et al., 2015). GLUT5 could therefore secrete glucose into the lumen under fasting conditions. The magnitude and significance of such transport would of course depend on glucose affinity, transport capacity, and cytosol-lumen gradients.

At higher luminal glucose concentrations, apical GLUT2 may lead to an increase in glucose uptake. However, this depends on a number of factors. If the source of apical GLUT2 is translocation from the basolateral membrane, glucose efflux capacity into the interstitium decreases. Consequently net glucose absorption and blood glucose concentration are reduced. If apical GLUT2 derives from a cytosolic source without reducing basolateral GLUT2, the maximum increase in glucose uptake (about 30% from baseline for a luminal glucose concentration of 50 mM) occurs when the blood flow rate is high and the inlet blood glucose is low. In reality, blood flow and glucose are likely to increase in parallel and so this condition represents an upper limit on the enhancement of glucose uptake by apical GLUT2. In all cases considered, an increase in SGLT1 density is more effective at increasing glucose absorption compared to an increase in apical GLUT2 density.

**Figure 9:**
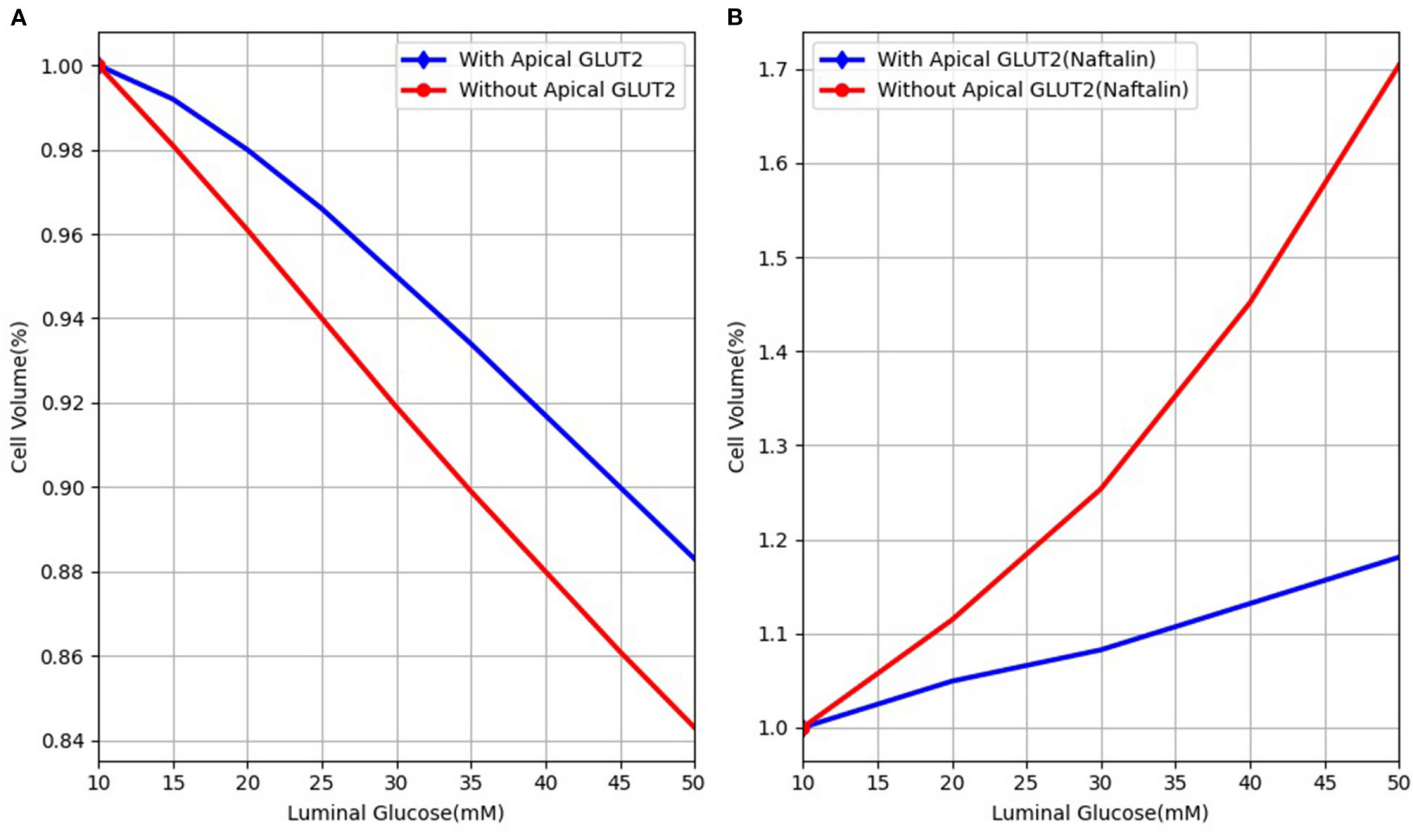
Cell volume behaviour in response to different luminal glucose levels in **(A)** our model and **(B)** Naftalin (2014) model. Values are normalised to baseline values.

The model presented here differs from previous cellular models (Thorsen et al., 2014; Afshar et al., 2019) by considering non-isotonic transport. It is, therefore, able to model more realistic scenarios, such as those encountered in experimental manipulations. Our model is similar in some respects to the Naftalin (2014) model that also simulates non-isotonic transport and includes apical GLUT2 transporters. Similar to that model, we found that increasing apical GLUT2 expression has a limited effect on increasing glucose absorption and may even decrease it by reducing the efflux capacity at the basolateral membrane. The two models also agree that increased blood flow is important for increased glucose absorption. One important point of difference is the cell volume change of the enterocyte when presented with a luminal glucose stimulus. The predictions of the two models are shown in Figure 9. In our model, the cell shrinks as water is transported across the apical membrane into the hyperosmolar lumen. The presence of apical GLUT2 reduces the magnitude of the decrease. In Naftalin's model, cell volume increases with luminal glucose [Figure 9B, reproduced from (Figure 2C) of his article]. This difference is likely due to the different parameter values used in the models. For the conditions of Figure 9B, cytosolic glucose concentrations exceed luminal and interstitial glucose levels due to uphill transport by SGLT1 and thus water enters the cell from the extracellular compartments. In our model, the cell is hypo-osmotic to the lumen for the conditions simulated in Figure 9. However, conditions similar to Naftalin's model can be obtained by increasing SGLT1 levels and tuning the apical to basolateral GLUT2 ratio as seen in Figure 8.

In conclusion, a model based analysis of glucose uptake by the enterocyte reveals that transport across apical GLUT2 is sensitive to the prevailing conditions of luminal glucose, blood flow, inlet blood glucose concentration, and the source of the apical GLUT2. In contrast, uptake by SGLT1 is less sensitive to these factors and its kinetic response serves to reduce variations in glucose uptake as these factors change. In addition, short term upregulation of SGLT1 is more effective than a similar upregulation of apical GLUT2 at responding to an increase in luminal glucose concentration.

## Data Availability Statement

The original contributions presented in the study are included in the article/Supplementary Material, further inquiries can be directed to the corresponding author/s.

## Author Contributions

NA, SS, DN, PH, and VS contributed to the concept and design of the study. NA implemented, checked, and validated the model and interpreted the results. NA, VS, and SS and wrote the first draft of the manuscript. All authors contributed to the manuscript revision, read, and approved the submitted version.

## Funding

NA was funded by a doctoral scholarship from the Riddet Centre of Research Excellence. VS acknowledges funding from RSNZ Marsden (contract UOA1411).

## Conflict of Interest

The authors declare that the research was conducted in the absence of any commercial or financial relationships that could be construed as a potential conflict of interest.

## Publisher's Note

All claims expressed in this article are solely those of the authors and do not necessarily represent those of their affiliated organizations, or those of the publisher, the editors and the reviewers. Any product that may be evaluated in this article, or claim that may be made by its manufacturer, is not guaranteed or endorsed by the publisher.

## References

[B1] Affleck J. A. Helliwell P. A. Kellett G. L. (2003). Immunocytochemical detection of glut2 at the rat intestinal brush-border membrane. J. Histochem. Cytochem. 51, 1567–1574. 10.1177/002215540305101116 14566028 PMC3957565

[B2] Afshar N. Safaei S. Nickerson D. P. Hunter P. Suresh V. (2019). Computational modelling of glucose uptake in the enterocyte. Front. Physiol. 10:380. 10.3389/fphys.2019.00380 31031632 PMC6473069

[B3] Argyropoulos C. Rondon-Berrios H. Raj D. S. Malhotra D. Agaba E. I. Rohrscheib M.. (2016). Hypertonicity: pathophysiologic concept and experimental studies. Cureus 8:e596. 10.7759/cureus.596 27382523 PMC4895078

[B4] Au A. Gupta A. Schembri P. Cheeseman C. I. (2002). Rapid insertion of glut2 into the rat jejunal brush-border membrane promoted by glucagon-like peptide 2. Biochem. J. 367(Pt 1):247. 10.1042/bj20020393 12095416 PMC1222871

[B5] Barrett K. E. Keely S. J. (2015). Chapter 24: Electrolyte secretion and absorption in the small intestine and colon. Yamada's Textbook Gastroenterol. 420–449. 10.1002/9781118512074.ch2425855820

[B6] Cheeseman C. I. (1997). Upregulation of sglt-1 transport activity in rat jejunum induced by glp-2 infusion *in vivo*. Am. J. Physiol. Regul. Integr. Compar. Physiol. 273, R1965–R1971. 10.1152/ajpregu.1997.273.6.R1965 9435650

[B7] Cohen M. Kitsberg D. Tsytkin S. Shulman M. Aroeti B. Nahmias Y. (2014). Live imaging of glut2 glucose-dependent trafficking and its inhibition in polarized epithelial cysts. Open Biol. 4:140091. 10.1098/rsob.140091 25056286 PMC4118605

[B8] Collin J. Kelly K. A. Phillips S. F. (1978). Increased canine jejunal absorption of water, glucose, and sodium with intestinal pacing. Am. J. Dig. Dis. 23, 1121–1124. 10.1007/BF01072888 736018

[B9] Corpe C. P. Basaleh M. M. Affleck J. Gould G. Jess T. J. Kellett G. L. (1996). The regulation of glut5 and glut2 activity in the adaptation of intestinal brush-border fructose transport in diabetes. Pflügers Archiv Eur. J. Physiol. 432, 192–201. 10.1007/s004240050124 8662294

[B10] Debnam E. Levin R. (1975). An experimental method of identifying and quantifying the active transfer electrogenic component from the diffusive component during sugar absorption measured *in vivo*. J. Physiol. 246, 181–196. 10.1113/jphysiol.1975.sp010885 1133782 PMC1309409

[B11] Ferraris R. P. (2001). Dietary and developmental regulation of intestinal sugar transport. Biochem. J. 360, 265–276. 10.1042/bj3600265 11716754 PMC1222226

[B12] Ferraris R. P. Diamond J. (1997). Regulation of intestinal sugar transport. Physiol. Rev. 77, 257–302. 10.1152/physrev.1997.77.1.257 9016304

[B13] Ferraris R. P. Yasharpour S. Lloyd K. Mirzayan R. Diamond J. M. (1990). Luminal glucose concentrations in the gut under normal conditions. Am. J. Physiol. Gastrointest. Liver Physiol. 259, G822–G837. 10.1152/ajpgi.1990.259.5.G822 2240224

[B14] Frizzell R. A. Nellans H. N. Rose R. C. Markscheid-Kaspi L. Schultz S. G. (1973). Intracellular cl concentrations and influxes across the brush border of rabbit ileum. Am. J. Physiol. Legacy Content 224, 328–337. 10.1152/ajplegacy.1973.224.2.328 4346845

[B15] Fullerton P. M. Parsons D. (1956). The absorption of sugars and water from rat intestine *in vivo. Exp. Physiol*. 41, 387–397. 10.1113/expphysiol.1956.sp0012103763805

[B16] Gouyon F. Caillaud L. Carriere V. Klein C. Dalet V. Citadelle D.. (2003). Simple-sugar meals target glut2 at enterocyte apical membranes to improve sugar absorption: a study in glut2-null mice. J. Physiol. 552, 823–832. 10.1113/jphysiol.2003.049247 12937289 PMC2343460

[B17] Granger D. Richardson P. Kvietys P. Mortillaro N. (1980). Intestinal blood flow. Gastroenterology 78, 837–863. 10.1016/0016-5085(80)90692-7 6101568

[B18] Kararli T. T. (1995). Comparison of the gastrointestinal anatomy, physiology, and biochemistry of humans and commonly used laboratory animals. Biopharm. Drug Disposit. 16, 351–380. 10.1002/bdd.2510160502 8527686

[B19] Karasov W. H. (2017). Integrative physiology of transcellular and paracellular intestinal absorption. J. Exp. Biol. 220, 2495–2501. 10.1242/jeb.144048 28724701

[B20] Kellett G. L. Helliwell P. A. (2000). The diffusive component of intestinal glucose absorption is mediated by the glucose-induced recruitment of glut2 to the brush-border membrane. Biochem. J. 350, 155–162. 10.1042/bj3500155 10926839 PMC1221237

[B21] Koepsell H. (2020). Glucose transporters in the small intestine in health and disease. Pflgers Arch. Eur. J. Physiol. 472. 10.1007/s00424-020-02439-5 32829466 PMC7462918

[B22] Kuzmuk K. N. Swanson K. S. Tappenden K. A. Schook L. B. Fahey G. C. (2005). Diet and age affect intestinal morphology and large bowel fermentative end-product concentrations in senior and young adult dogs. J. Nutr. 135. 10.1093/jn/135.8.1940 16046720

[B23] Lane J. S. Whang E. E. Rigberg D. A. Hines O. J. Kwan D. Zinner M. J.. (1999). Paracellular glucose transport plays a minor role in the unanesthetized dog. Am. J. Physiol. Gastrointest. Liver Physiol. 276, G789–G794. 10.1152/ajpgi.1999.276.3.G789 10070058

[B24] Lowe A. G. Walmsley A. R. (1986). The kinetics of glucose transport in human red blood cells. Biochim. Biophys. Acta Biomembr. 857, 146–154. 10.1016/0005-2736(86)90342-1 3707948

[B25] McCance R. A. Madders K. (1930). The comparative rates of absorption of sugars from the human intestine. Biochem. J. 24, 795. 10.1042/bj0240795 16744419 PMC1254520

[B26] Naftalin R. J. (2014). Does apical membrane glut2 have a role in intestinal glucose uptake? F1000Research 3:304. 10.12688/f1000research.5934.1 25671087 PMC4309173

[B27] Nellans H. N. Frizzell R. A. Schultz S. G. (1973). Coupled sodium-chloride influx across the brush border of rabbit ileum. Am. J. Physiol. Legacy Content 225, 467–475. 10.1152/ajplegacy.1973.225.2.467 4352899

[B28] Nellans H. N. Schultz S. G. (1976). Relations among transepithelial sodium transport, potassium exchange, and cell volume in rabbit ileum. J. Gen. Physiol. 68, 441–463. 10.1085/jgp.68.4.441 993767 PMC2228441

[B29] NIfHa,C E. (2012). Type 2 diabetes: prevention in people at high risk. NICE Guideline (PH38).

[B30] Nomura N. Verdon G. Kang H. J. Shimamura T. Nomura Y. Sonoda Y.. (2015). Structure and mechanism of the mammalian fructose transporter glut5. Nature 10.4751038/nature14909 26416735 PMC4618315

[B31] Pappenheimer J. (1987). Physiological regulation of transepithelial impedance in the intestinal mucosa of rats and hamsters. J. Membr. Biol. 100, 137–148. 10.1007/BF02209146 3430570

[B32] Pappenheimer J. Reiss K. (1987). Contribution of solvent drag through intercellular junctions to absorption of nutrients by the small intestine of the rat. J. Membr. Biol. 100, 123–136. 10.1007/BF02209145 3430569

[B33] Parent L. Supplisson S. Loo D. D. Wright E. M. (1992). Electrogenic properties of the cloned na+/glucose cotransporter: Ii. a transport model under nonrapid equilibrium conditions. J. Membr. Biol. 125, 63–79. 10.1007/BF00235798 1294062

[B34] Pfortmueller C. A. Uehlinger D. von Haehling S. Schefold J. C. (2018). Serum chloride levels in critical illnessthe hidden story. Intens. Care Med. Exp. 6:10. 10.1186/s40635-018-0174-5 29654387 PMC5899079

[B35] Pierro A. Coppi P. D. Eaton S. (2012). Chapter 6-neonatal physiology and metabolic considerations, in Pediatric Surgery, 7th Edn, ed Coran A. G. (Philadelphia, PA: Mosby), 89–107.

[B36] Posencheg M. A. Evans J. R. (2012). Chapter 31-acid-base, fluid, and electrolyte management, in Avery's Diseases of the Newborn, 9th Edn, eds Gleason C. A. Devaskar S. U. (Philadelpia, PA: W. B. Saunders), 367–389.

[B37] Rieke W. O. Everett N. B. (1957). Effect of pentobarbital anesthesia on the blood values of rat organs and tissues. Am. J. Physiol. Legacy Content 188, 403–408. 10.1152/ajplegacy.1957.188.2.403 13411224

[B38] Robinson J. W. L Menge,' H. Sepulveda F. V. Mirkovitch V. (1977). Functional and structural characteristics of the jejunum and ileum in the dog and the rat. Digestion 15, 188–199. 10.1159/000198003 844673

[B39] Rose R. C. Schultz S. G. (1971). Studies on the electrical potential profile across rabbit ileum. J. Gen. Physiol. 57, 639–663. 10.1085/jgp.57.6.639 5576764 PMC2203124

[B40] Sharp P. A. Debnam E. S. Srai S. K. (1996). Rapid enhancement of brush border glucose uptake after exposure of rat jejunal mucosa to glucose. Gut 39, 545–550. 10.1136/gut.39.4.545 8944563 PMC1383267

[B41] Shimada T. Hoshi T. (1987). Role of na+/h+ antiport in intracellular ph regulation by rabbit enterocytes. Biochim. Biophys. Acta Biomembr. 901, 265–272. 10.1016/0005-2736(87)90123-4 3038188

[B42] Stearns A. T. Balakrishnan A. Rhoads D. B. Tavakkolizadeh A. (2010). Rapid upregulation of sodium-glucose transporter sglt1 in response to intestinal sweet taste stimulation. Ann. Surg. 251, 865–871. 10.1097/SLA.0b013e3181d96e1f 20395849 PMC4123655

[B43] Thorsen K. Drengstig T. Ruoff P. (2014). Transepithelial glucose transport and na+/k+ homeostasis in enterocytes: an integrative model. Am. J. Physiol. Cell Physiol. 307, C320–C337. 10.1152/ajpcell.00068.2013 24898586 PMC4137142

[B44] Wertheimer E. (1934). Phloridzinwirkung auf die zuckerresorption. Pflüger's Archiv für die gesamte Physiol. Mensch. der Tiere 233, 514–528. 10.1007/BF01751462

[B45] Wright E. M. Loo D. D. Hirayama B. A. (2011). Biology of human sodium glucose transporters. Physiol. Rev. 91, 733–794. 10.1152/physrev.00055.2009 21527736

[B46] Zeuthen T. Zeuthen E. MacAulay N. (2007). Water transport by glut2 expressed in xenopus laevis oocytes. J. Physiol. 579, 345–361. 10.1113/jphysiol.2006.123380 17158169 PMC2075391

