# Supplementary material for: Computational Modelling of Glucose Uptake by SGLT1 and Apical GLUT2 in the Enterocyte
Source: Front Physiol. 2021 Dec 7;12:699152. doi: 10.3389/fphys.2021.699152 (PMC8688934; doi:10.3389/fphys.2021.699152)
Supplement: Supplementary file 1 [file Data_Sheet_1.PDF]

Supplementary Material: Computational Modelling of Glucose Uptake in the Enterocyte

Nima Afshar [1,*], Soroush Safaei [1], David Nickerson [1], Peter Hunter [1] and Vinod Suresh [1,2]

*Correspondence:
Vinod Suresh
v.suresh@auckland.ac.nz

1 MODEL EQUATIONS

Membrane Potential

Based on the conservation of charge, summation of all ionic currents through the apical or basolateral membrane divided by the cell capacitance gives the membrane potential. It includes all the currents via electrogenic ion channels and paracellular pathway. Electroneutral transporters do not play any role in the net flux of charge. A positive ion entering the cell at either the apical or basolateral membrane is treated as a positive current

$$C\frac{dv_{mc}}{dt} = (I_{Na_{SGLT1}} + I_{BK} + I_{ENaC} + I_{CFTR} + I_P^{Na} + I_P^{K} + I_P^{Cl}) \tag{S1}$$

$$C\frac{dv_{sc}}{dt} = (I_{KCNQ} + I_{CLC2} + I_{K_{NaK}} - I_{Na_{NaK}} + I_{NBC_{Na}} - I_{NBC_{HCO3}} - I_P^{Na} - I_P^{K} - I_P^{Cl}) \tag{S2}$$

Ionic balance equation

Intracellular ion concentrations are calculated based on the summation of all the inlet and outlet fluxes divided by the cell volume. The flux J_m of species m is equal to the current I_m of that species divided by the product of ion's valence and the Faraday number. The equation for pH uses the buffering capacity β (mM/pH unit). Expressions for the individual currents and fluxes are found the in the references cited in Table 1.

$$\frac{d[Na]_i}{dt} = (\frac{1}{v_{cell}})(J_{Na_{SGLT1}} - J_{Na_{NaK}} + J_{ENaC} + J_{NaKCC} + J_{Na_{NBC}} + J_{Na_{NHE3}} - [Na]_i(\frac{dv_{cell}}{dt})) \tag{S3}$$

$$\frac{d[glucose]_i}{dt} = (\frac{1}{v_{cell}})(J_{m_{GLUT2}} + J_{Gl_{SGLT1}} - J_{s_{GLUT2}} - [glucose_i](\frac{dv_{cell}}{dt})) \tag{S4}$$

$$\frac{d[K]_i}{dt} = (\frac{1}{v_{cell}})(J_{K_{NaK}} + J_{NaKCC} + J_{KCNQ} + J_{BK} - [K]_i(\frac{dv_{cell}}{dt})) \tag{S5}$$

$$\frac{d[Cl]_i}{dt} = (\frac{1}{v_{cell}})(2 \times J_{NaKCC} + J_{Cl_{AE1}} - J_{CFTR} - J_{CLC2} - [Cl]_i(\frac{dv_{cell}}{dt})) \tag{S6}$$

$$\frac{dpH_i}{dt} = (\frac{\rho}{\beta})(J_{HCO3_{NBC}} - J_{H_{NHE3}} - J_{HCO3_{AE1}}) \tag{S7}$$

Water flux

Water is transported through the water channel aquaporin, AQP, and results in cell volume changes (figure **??**). The cell is considered as a cuboidal shape with a constant area at the apical and basolateral surface. The volume of the cell, v_{cell}, is therefore calculated based on water flux through both membranes.

$$\frac{dv_{cell}}{dt} = V_W(J_s^w - J_m^w) \tag{S8}$$

V_W is the partial molar volume of water. Water flux J^w is usually governed by the osmotic gradient and hydrostatic pressure gradient, however in this model we assumed there to be no hydrostatic pressure gradient and osmotic gradient was taken as the only driving force of J^w.

$$\begin{aligned} J_m^w &= L_A A[\sum[n]_m + (\frac{\psi_m}{v_{cell}}) - \sum[n]_i - (\frac{\chi_i}{v_{cell}})] \\ J_s^w &= L_B A[\sum[n]_i + (\frac{\chi_i}{v_{cell}}) - \sum[n]_s] \\ J_P^w &= L_P A[\sum[n]_m + (\frac{\psi_m}{v_{cell}}) - \sum[n]_s] \end{aligned} \tag{S9}$$

where L_A and L_B are the hydraulic conductances of the apical and basolateral membranes, and L_P is the conductance via the paracellular pathway. χ^- shows the negatively charged impermeable moles inside the cell, and ψ represents uncharged impermeable species. [n] is the sum of concentrations of all ions (Na^+, Cl^-, K^+, H^+,$HCO3^-$, Ca^+ and glucose in each compartment).
